# Neutralizing anti-HIV antibodies develop in a humanized mouse model of HIV-1 infection

**DOI:** 10.1186/1742-4690-9-S2-P60

**Published:** 2012-09-13

**Authors:** E Seung, A Dugast, T Dudek, H Mattoo, V Vrbanac, T Tivey, T Murooka, A Cariappa, AD Luster, S Pillai, AM Tager

**Affiliations:** 1 Massachusetts General Hospital, Charlestown, MA, USA; 2 Ragon Institute of MGH, MIT, and Harvard, Charlestown, MA, USA; 3 MGH Cancer Center, Charlestown, MA, USA

## Background

In BLT (bone marrow-liver-thymus) humanized mice, human thymocytes are educated by autologous human thymic tissue, resulting in functional human T cells capable of rapidly selecting for CTL escape mutations in HIV. In contrast, limitations to B cell maturation have been noted. But despite this, we show for the first time that HIV infected BLT mice can produce class-switched anti-HIV antibodies with neutralizing activities.

## Methods

Humanized BLT mice were generated by transplanting irradiated NOD-scid/IL2rgnull (NSG) mice with fetal thymus and liver fragments and then injecting them with autologous human CD34+ stem cells. BLT mice were then infected with HIV_JRCSF_ and bled at various time-points. HIV neutralizing activity was measured using Tat-induced luciferase reporter TZM-bl cells.

## Results

Human transitional B cells were present in greater frequencies in BLT mice than adult humans. Most of these cells had a T1 phenotype in the blood and spleen. But despite this B cell maturation defect, class-switched IgG Abs against various HIV proteins were detected by Western Blot in HIV-infected BLT mice. Using ELISA to determine anti-p24 IgG Ab titers, Abs were present as early as 8 weeks post infection (p.i.), with peak Ab titers seen after 15 weeks. One infected mouse demonstrated a peak titer similar to that seen in a chronically infected human. Finally, plasma samples from infected BLT mice after 22 weeks p.i. demonstrated neutralizing activities against the challenge virus. Average IC50 neutralizing titers in these mice were similar to those from infected human samples.

## Conclusion

The ability of humanized BLT mice to generate functional humoral immune responses may be further improved by strategies to improve their B cell maturation, which will further improve the potential of these mice to become a model system to study candidate HIV vaccines and therapies.

